# On the design and performance analysis of wristband MIMO/diversity antenna for smart wearable communication applications

**DOI:** 10.1038/s41598-021-01326-y

**Published:** 2021-11-09

**Authors:** Thennarasi Govindan, Sandeep Kumar Palaniswamy, Malathi Kanagasabai, Thipparaju Rama Rao, M. Gulam Nabi Alsath, Sachin Kumar, Sangeetha Velan, Mohamed Marey, Apeksha Aggarwal

**Affiliations:** 1 Department of Electronics and Communication Engineering, SRM Institute of Science and Technology, Kattankulathur, 603203 India; 2 Department of Electronics and Communication Engineering, Anna University, Chennai, 600025 India; 3 Department of Electronics and Communication Engineering, Sri Sivasubramania Nadar (SSN) College of Engineering, Chennai, 603110 India; 4 Department of Research and Development, FLDEC Systems Private Limited, Chennai, 600032 India; 5 Smart Systems Engineering Laboratory, College of Engineering, Prince Sultan University, Riyadh, 11586 Saudi Arabia; 6 Department of Computer Science Engineering and Information Technology, Jaypee Institute of Information Technology, Noida, 201309 India

**Keywords:** Nanoscience and technology, Engineering, Biomedical engineering, Electrical and electronic engineering

## Abstract

The design of a silicone rubber-based wristband wearable antenna exploiting pattern diversity is presented in this paper. The wristband diversity antenna consists of four identical antenna elements with an inter-element spacing of 0.68*λ*_0_, where *λ*_0_ is the lower cut-off wavelength. A modified trapezoidal-shaped radiator with a rectangular ground structure is used to achieve ultra-wide bandwidth. The proposed multiple-input-multiple-output (MIMO)/diversity antenna covers a frequency range of 2.75–12 GHz. The antenna element offers a radiation efficiency of 89.3% and a gain of 3.41 dBi. The size of the wristband diversity antenna is 1.1*λ*_0_ × 18.4*λ*_0_ × 0.18*λ*_0_. The diversity performance characteristics of the prototype antenna are examined, with the envelope correlation coefficient (ECC) < 0.18, apparent diversity gain (ADG) > 9.5, effective diversity gain (EDG) > 9.5, mean effective gain (MEG) < 1 dB, total active reflection coefficient (TARC) < − 10 dB, and channel capacity loss (CCL) < 0.1  bits/s/Hz over the entire operating band. The specific absorption rate (SAR) of the proposed wristband antenna is analyzed to determine its radiation exposure on the human body, and the results show that the values are less than 0.02 W/kg.

## Introduction

Wearable technology is becoming increasingly popular due to its numerous applications in health care, navigation, security, smart home, and defense. It can be used in both rigid and flexible devices^1^. Materials used as rigid substrates include FR-4 and Taconic^2^. In the literature, flexible substrates such as polymer, paper, PDMS, Ninja Flex, and Kapton were suggested for wearable applications^3–5^. Jeans is widely used for textile applications^6^. Conformal antennas are strongly recommended for maintaining mechanical flexibility while retaining their original shape and characteristics^7^. In the wearable concept, one should be aware of radiation exposure from the antenna while in close contact with the human body. The radiation effect should be as small as possible so that it does not harm human tissues. The radiation level can be determined using a specific absorption rate (SAR) analysis, which will be covered in detail in section “SAR performance”. Monopole antennas are preferred for on-body communication to avoid radiation exposure to the body^8^. They also have an extremely broad bandwidth^9^. ISM bands are frequently used for wearable applications in most cases. However, due to the narrow bandwidth coverage of the ISM band frequencies, they cannot be used for high data rate transmission. To overcome the limitations caused by narrow bandwidth, such as lower data rate and multipath fading, the Federal Communications Commission (FCC) assigned an unlicensed frequency range of 3.1–10.6 GHz, popularly known as ultra-wideband (UWB)^10^.

In recent years, the UWB has become very popular for medical applications due to its high data transfer speed and low energy consumption. UWB is commonly used in cardiology, pneumonology, and neurology systems for patient motion and vital sign monitoring, detecting not only macro motions but also minute movements. The 8–12 GHz frequency range is recommended for medical therapeutic applications. One important reason for using UWB in the medical industry is that it does not cause interference when combined with the medical therapy radiated signal. UWB radars are used to protect the pharmaceutical storage chambers. UWB is also recommended for underwater medicine, sports medicine, military medicine, and emergency medicine measurements^11^. Multipath fading is a serious problem in wireless communication as it decreases signal strength during data transfer^12^. There are various schemes for reducing multipath interference. One of the most common is spatial diversity, which employs two or more antennas to enhance the performance and reliability of a radio link^13^. In the multiple-input-multiple-output (MIMO)/diversity technique, multiple receivers are used to receive the same transmitted signal, and the best among them is processed to obtain the information^14^. However, the main issue with multiple antenna elements is the coupling between them. Because the increasing mutual coupling will lead to the degradation of diversity nature of the antenna^15^. Various techniques, such as electromagnetic band gap (EBG), split ring resonator (SRR), defected ground structure (DGS), and the introduction of decoupling elements, are used to improve inter-element isolation^16,17^. The positioning of the antenna elements is also important in order to avoid mutual coupling. The mutual coupling will be stronger if the antenna elements are placed close together^18^. However, placing them too far apart results in a large antenna size. In general, a minimum spacing of *λ*/2 between antenna elements is recommended to compensate mutual coupling. However, *λ*/2 spacing is not preferred in miniaturized circuits/modules^19^.

In this paper, a wearable quad-port MIMO/diversity antenna is designed and developed. The proposed antenna is made of a silicone wristband that is commonly available in the market at a low cost. Depending on the application, the proposed antenna can be used to monitor children and patients, ensuring user safety. In section “Antenna design”, the properties of the substrate material and the antenna design are described in detail. Section “Results and discussion” presents the results and discussion. The diversity performance of the proposed antenna is discussed in section “Diversity metrics”. SAR analysis is given in section “SAR performance” and section “Conclusion” closes the conclusion part.

## Antenna design

### Antenna element

**Figure 1:**
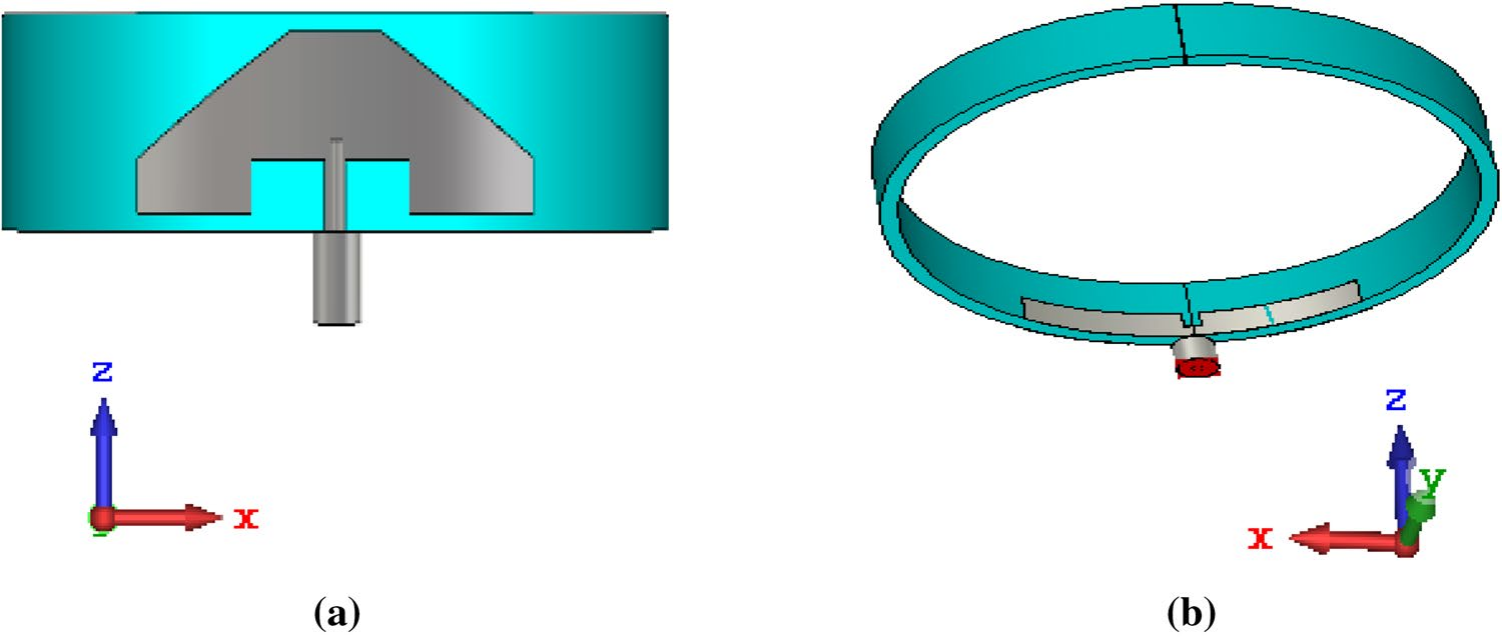
Proposed antenna (**a**) front view and (**b**) rear view.

**Figure 2:**
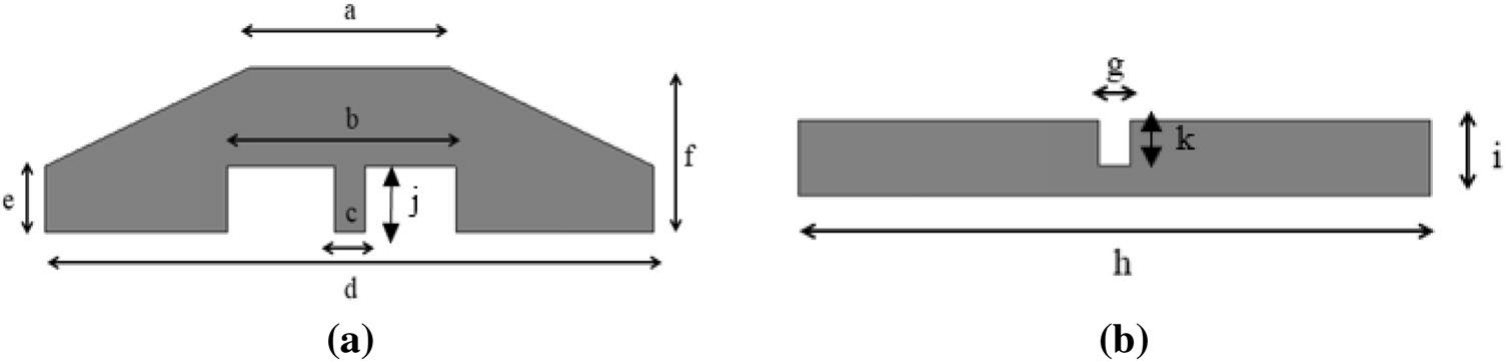
Dimensions of the wristband antenna (**a**) radiator (**b**) ground plane (*a *= 13  mm, *b *= 15 mm, *c *= 2 mm, *d *=4 0 mm, *e *= 4 mm, *f *= 12 mm, *g *= 2 mm, *h *= 40 mm, *i *= 5 mm, *j *= 4 mm, *k *= 3 mm).

The geometry of the proposed monopole antenna element is depicted in Fig. 1. It is designed on the silicone rubber substrate material of relative permittivity (*ɛ*_*r*_) of 4 and loss tangent (tan *δ*) of 0.358^19^. Since the wristband is made of silicone rubber, it is chosen as the substrate material. Also, this material shows resistive nature when exposed to the outside environment. It can also withstand extreme temperatures ranging from − 100 to 250 °C^20^. The antenna element functions in the UWB (3.1–10.6 GHz) frequency range. The dimensions of the radiating patch and ground plane are shown in Fig. 2. The wristband is available in various sizes for different age groups, such as for kids and adults. For the proposed work, an adult size wristband with a circumference of 202 mm and dimension of 2.08*λ*_0_, where *λ*_0_ corresponds to the lowest operating wavelength, is chosen. The height and thickness of the substrate are 12 mm and 2 mm, respectively. The total area of the antenna element on the wristband is approximately 10 × 40 mm^2^ (0.09*λ*_0_ × 0.36*λ*_0_).

### Design process

For monopole antennas, the lower band-edge frequency (*f*_1_) of the UWB frequency band is calculated using Eq. (1)^21^

1 $${ f}_{1}=\frac{7.2}{(l+r+p)\times k}$$

where *p* is the distance between the radiator and the ground plane, *l* and *r* are the height and width of the monopole antenna, respectively, and *k* is the fourth root of the effective dielectric constant. For the proposed wristband antenna, Eq. (1) is rewritten as

2 $${f}_{1}=\frac{7.2}{(1.21\pi \left[\left(d+f\right)\right]+p)\times k}$$

The expression (*l* + *r*) is equivalent to the term $1.21\pi$ (*d* + *f*), which corresponds to the perimeter of the monopole radiator. The notations *d* and *f* represent the semi-width and semi-length of the proposed radiator.

**Figure 3:**
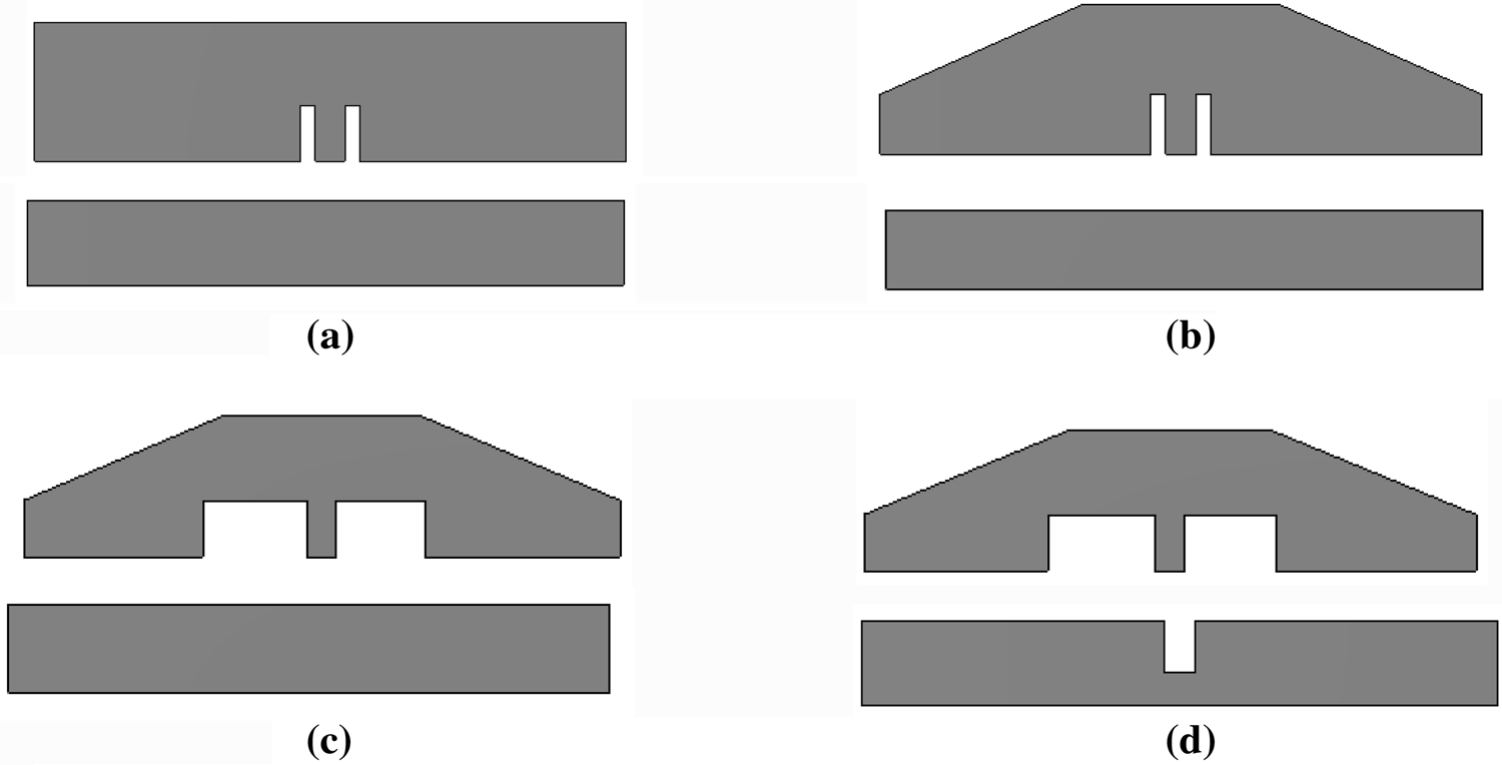
Evolution stages of the wristband antenna (**a**) antenna 1, (**b**) antenna 2, (**c**) antenna 3 and (**d**) antenna 4.

**Figure 4:**
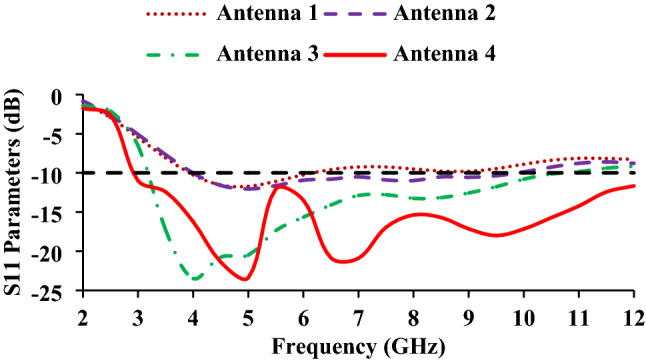
Reflection coefficients of the evolution stages.

The antenna design process begins with a rectangular radiator and a ground plane of length of 6 mm, as shown in Fig. 3a. However, the antenna 1 has poor impedance matching. In evolution step 2, both sides of the radiator are tapered (Fig. 3b), and the length of the ground plane is reduced to 5 mm. Tapering is a design technique that shifts the resonance to the right or left by varying the physical length of the antenna. In this work, the electrical length of the radiator is shortened, and as a result, the frequency is shifted to the right (4–9.8 GHz), as shown in Fig. 4. In evolution stage 3, the slot width near the feed line is increased (Fig. 3c)^22^, to shift the resonance to the left side and achieve a wider bandwidth of 3.23–10.5 GHz. In evolution stage 4, a rectangular slot is loaded on the ground plane (Fig. 3d), which improves the impedance matching and increases the resonating range of the antenna. The proposed design (antenna 4) has a wider bandwidth of 3–12 GHz (Fig. 4).

**Figure 5:**
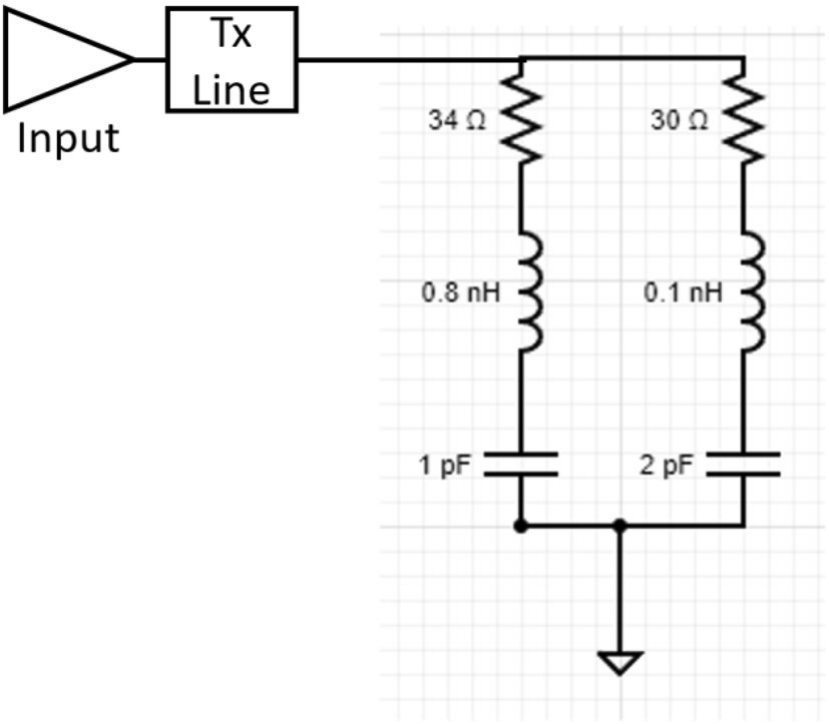
Equivalent circuit of the proposed antenna.

**Figure 6:**
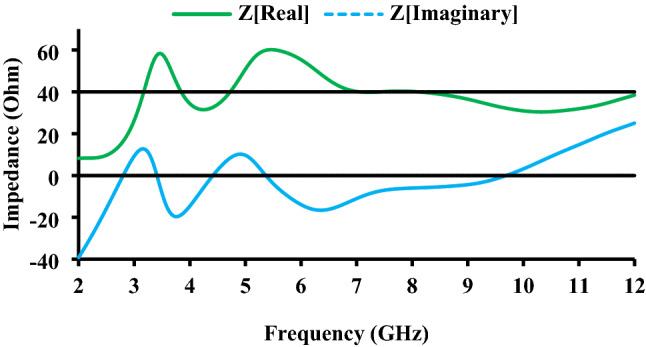
Impedance characteristics of the proposed antenna.

The equivalent circuit of the proposed antenna is shown in Fig. 5 for a better understanding of its performance. The equivalent circuit is drawn using the characteristics impedance (real and imaginary) curves, shown in Fig. 6. The *R*, *L*, and *C* components are determined by real and imaginary impedance values, and they are connected in parallel in the proposed antenna. The real and imaginary impedance curves play an important role in the arrangement of *R*, *L*, and *C*. If these curves run in opposite directions, either from low to high or from high to low, it is said to be a series connection^23^. When the curves move in the same direction, the *R*, *L*, and *C* are connected in parallel. At 3.5 GHz and 10.6 GHz, both the real and impedance curves move in the same direction, from high to low and from low to high, as shown in Fig. 6, therefore, the *R*, *L*, and *C* are connected in parallel. The transmission line is used for 50 Ω impedance matching. The resistor value is calculated using the real impedance curve.

### MIMO/diversity antenna

**Figure 7:**
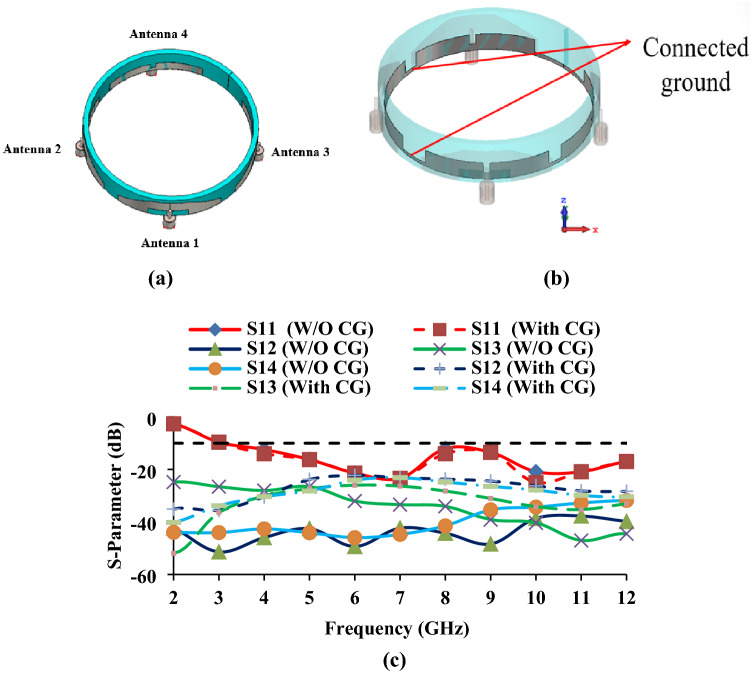
Proposed MIMO/diversity antenna (**a**) without connected ground plane, (**b**) with connected ground (CG) plane, (**c**) Simulated S-parameters.

**Figure 8:**
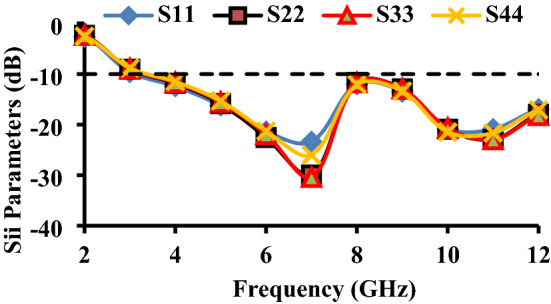
Simulated *S*_*ii*_*-*parameters of the proposed antenna.

Since the reflection coefficients of antenna 4 meet the desired response, it is extended for the development of a multi-antenna system. The proposed MIMO/diversity wristband antenna with^24^ and without common ground plane is shown in Fig. 7 and their corresponding S-parameter curves are shown in Fig. 8. It is found that the connected ground plane has no discernible effect on the performance of the proposed MIMO antenna. The mutual coupling curves with connected ground plane move slightly above the mutual coupling curves without connected ground plane, even though they are less than − 20 dB. Therefore, the connected ground plane has no effect on the proposed MIMO antenna performance. The polarization of the antenna is also checked, and it is found to be linearly polarized. In the wristband antenna, four antenna elements are placed horizontally to achieve pattern diversity. The distance between antenna elements (antennas 1, 2, 3, and 4) is kept at 7.5 mm (0.68 ${{\varvec{\lambda}}}_{0}).$ A proper spacing provides more than 20 dB isolation between the antenna elements.

## Results and discussion

### Reflection and transmission coefficients

**Figure 9:**
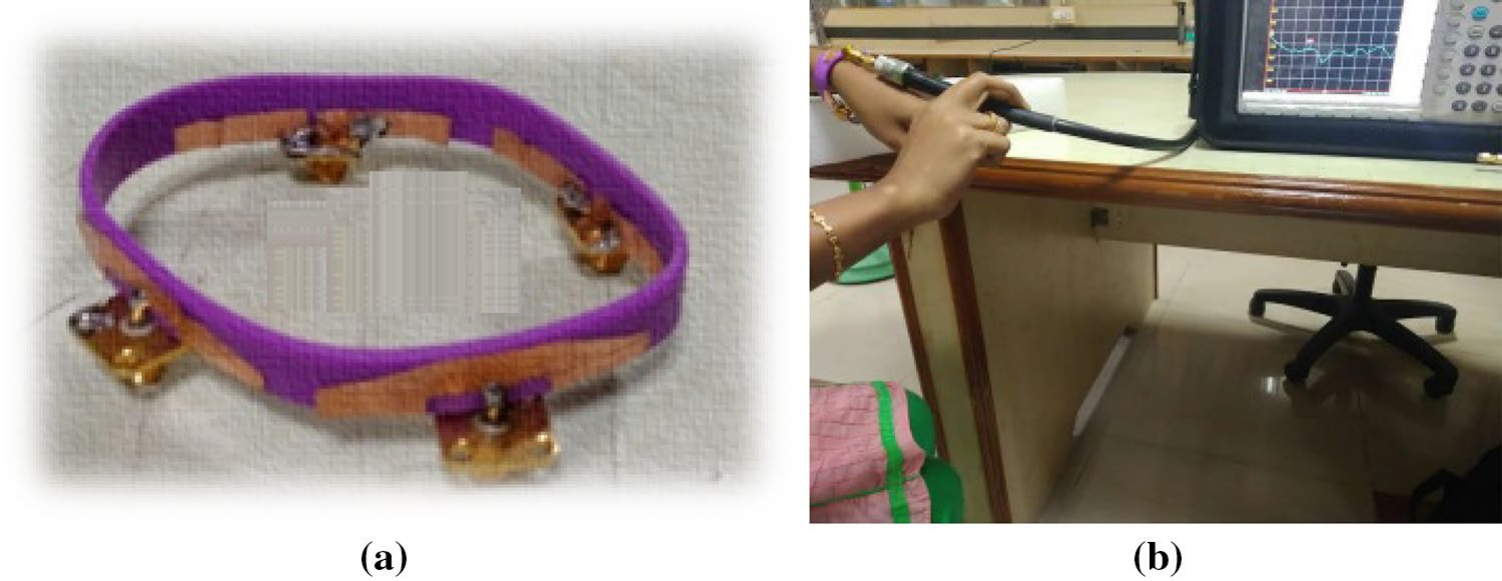
(**a**) Fabricated prototype and (**b**) antenna measurement using vector network analyzer.

**Figure 10:**
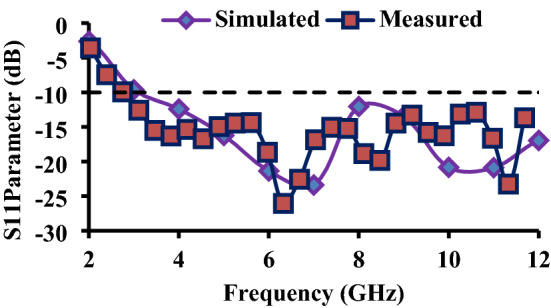
Measured and simulated *S*_11_ of the proposed antenna.

The reflection coefficients (${S}_{ii}$) of the antenna elements are shown in Fig. 8. The designed antenna is fabricated and tested using Anritsu MS2037C vector network analyzer. The fabricated prototype of the wristband antenna is shown in Fig. 9. Figure 10 depicts the simulated and measured reflection coefficients of the antenna element. The simulated |*S*_11_|≤ − 10 dB impedance bandwidth is 9 GHz (3–12 GHz), whereas the measured impedance bandwidth is 9.35 GHz (2.75–12 GHz). The simulated and measured results are found in the good agreement covering the entire UWB range. Since all four antenna elements are placed horizontally in one plane, their reflection coefficient curves are nearly identical.

**Figure 11:**
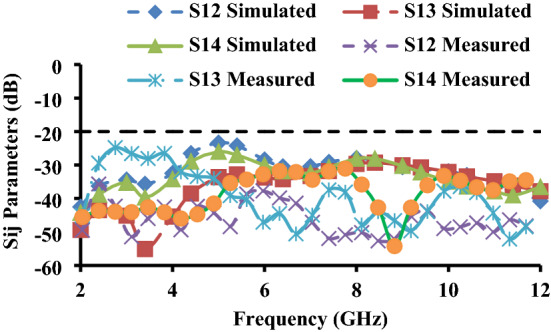
Measured and simulated *S*_*ij*_-parameters of the proposed antenna.

The transmission coefficients (${S}_{ij}$) are calculated at ports 2, 3, and 4, with antenna 1 considering as the reference antenna. When the other antenna elements (antennas 2, 3, and 4) were used as a reference, a similar response was observed. The measured and simulated ${S}_{ij}$-parameters are shown in Fig. 11. Isolation of more than 20 dB is observed for all four ports throughout the UWB range.

### Radiation characteristics

**Figure 12:**
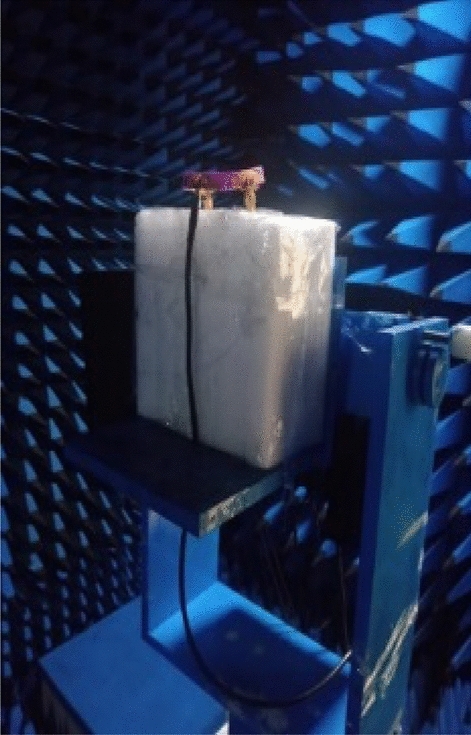
Anechoic chamber measurement setup.

**Figure 13:**
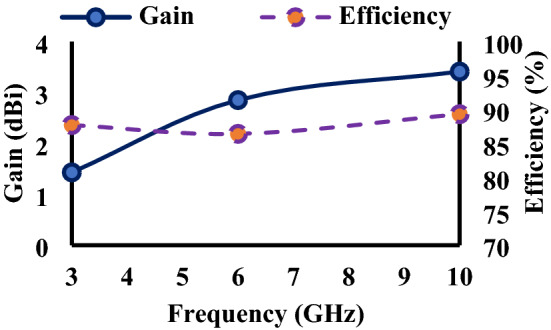
Gain and efficiency of the proposed antenna.

**Figure 14:**
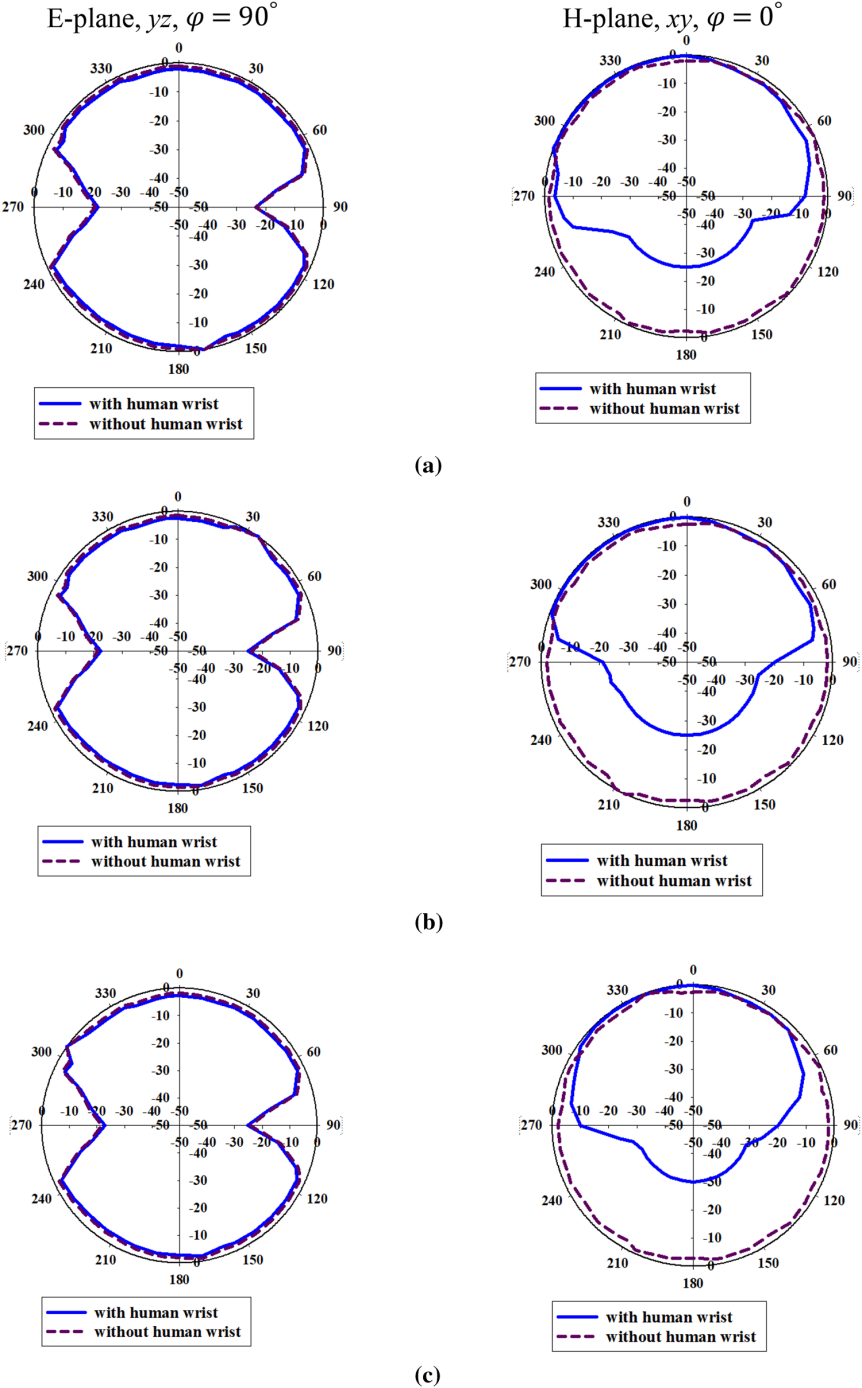
Radiation patterns of the proposed wristband antenna (**a**) 3 GHz, (**b**) 6 GHz and (**c**) 10 GHz.

The fabricated wristband antenna is measured in an anechoic chamber to obtain the far-field radiation patterns, with a standard horn antenna serving as the reference antenna. Figure 12 shows the anechoic chamber measurement setup with the proposed wristband antenna. Figure 13 represents the gain and efficiency curves of the wristband antenna. The measured gain values at 3 GHz, 6 GHz, and 10 GHz are 1.43 dBi, 2.85 dBi, and 3.41 dBi, respectively. The efficiency of the antenna is found to be 87.7% (3 GHz), 86.4% (6 GHz), and 89.3% (10 GHz). The radiation pattern comparison of the diversity antenna with and without human wrist is shown in Fig. 14 at three different frequencies (3 GHz, 6 GHz, and 10 GHz).

## Diversity metrics

### Envelope correlation coefficient (ECC)

ECC helps in the investigation of the correlation between radiation patterns. The ECC can be calculated using the far-field radiation patterns and the *S*-parameters. However, the far-field provides appropriate results. The formula used for calculating ECC using far-field is^8^

3 $${\rho }_{e}= \frac{{\left|\iint \left[\overrightarrow{{F}_{1}}\left(\theta ,\varphi \right).\overrightarrow{{F}_{2}}\left(\theta ,\varphi \right)\right]d\Omega \right|}^{2}}{\iint {\left|\overrightarrow{{F}_{1}}\left(\theta ,\varphi \right)\right|}^{2}d\Omega \iint {\left|\overrightarrow{{F}_{2}}\left(\theta ,\varphi \right)\right|}^{2 }d\Omega }$$

Equation (4) is used to calculate the ECC with *S*-parameters.

4 $${\rho }_{e}=\frac{{\left|{S}_{ii }^{*}{S}_{ij}+{S}_{ji}^{*}{S}_{jj}\right|}^{2}}{\left(1-{\left|{S}_{ii}\right|}^{2}-{\left|{S}_{ij}\right|}^{2}\right)\left(1-{\left|{S}_{ji}\right|}^{2}-{\left|{S}_{jj}\right|}^{2}\right)}$$

**Figure 15:**
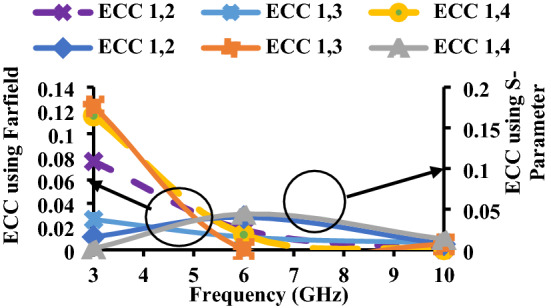
ECC of the proposed antenna.

Ideally, the ECC value should be zero, but practically, it is limited to < 0.5. The ECC curves of the proposed wristband antenna are shown in Fig. 15.

### Apparent diversity gain (ADG)

Diversity gain deals with the amount of transmitted power that can be reduced without any loss in diversity technique. It is related to ECC and is divided into ADG and EDG, which can be calculated using Eqs. (5) and (6). The ADG is calculated as

5 $${G}_{app}=10\times \sqrt{1-{{\rho }_{e}}^{2}}$$

### Effective diversity gain (EDG)

**Figure 16:**
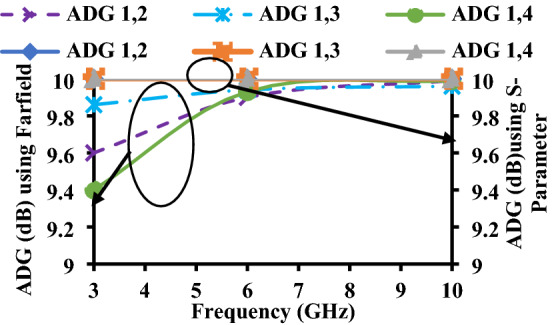
ADG of the proposed antenna.

**Figure 17:**
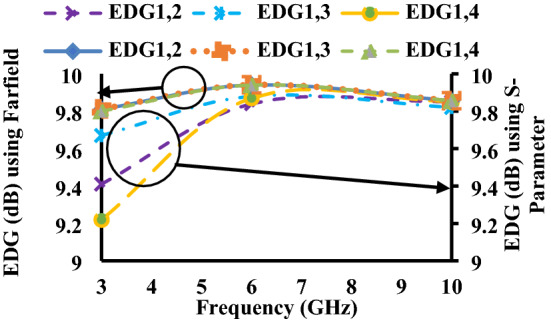
EDG of the proposed antenna.

The major difference between ADG and EDG is that ADG does not include radiation losses, whereas EDG includes radiation efficiency, which is multiplied with ADG. Due to some losses in EDG, its values are always less than the ADG^25^, as shown in Figs. 16 and 17.

6 $${G}_{eff}={\eta }_{total}\times {G}_{app}={\eta }_{total}\times 10\times \sqrt{1-{{\rho }_{e}}^{2}}$$

where

$${\eta }_{i,total}={\eta }_{i,rad}\left(1-{\sum }_{j=1}^{M}{\left|{S}_{ij}\right|}^{2}\right)$$

and $${\eta }_{i,rad}=\left(1-{\sum }_{j=1}^{M}\left|{S}_{ij}\right|\right)$$

### Mean effective gain (MEG)

**Figure 18:**
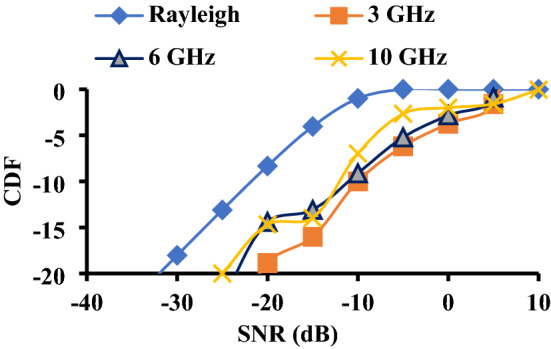
CDF of the proposed antenna.

MEG is the ratio of the average power received by the diversity antenna to the power received by an isotropic antenna. MEG values should be kept below 3 dB^26^. However, when calculated under three different scenarios isotropic (XPR = 0 dB), indoor (XPR = 1 dB), and outdoor (XPR = 5 dB), the MEG values are found to be 1 dB^8^. Another parameter used to validate diversity performance using MEG values is the cumulative distributive function (CDF). The CDF is calculated using Eq. (7), and the eigenvalues (*λ*) are obtained using Eq. (8). It is compared to the Rayleigh condition by taking the average of the signal-to-noise ratio (SNR). The plot between CDF and SNR is shown in Fig. 18.

7 $${P}_{MRC }\left(\gamma \le X\right)=1-\sum_{i=1}^{N}\left(\frac{{\lambda }_{i}^{N-1 }{e}^{-\frac{X}{{\lambda }_{i}}}}{\prod_{i\ne j}^{N}{\lambda }_{i}-{\lambda }_{j}}\right)$$

8 $${\Lambda }_{MRC}={\rho }_{e}\sqrt{{MEG}_{i}{MEG}_{j}}$$

where *x* denotes SNR, *N* represents the number of antennas,${\Lambda }_{MRC}$ is signal covariance matrix, and MRC is maximum ratio combining.

### Total active reflection coefficient (TARC)

It is defined as the ratio of the square root of total reflected power to the square root of total incident power^27^. For a multi-port antenna system, TARC can be calculated using the following equation

9 $$TARC \left({\Gamma }_{a}^{t}\right)= \frac{\sqrt{{\sum }_{i=1}^{N}{\left|{b}_{i}\right|}^{2}}}{\sqrt{{\sum }_{i=1}^{N}{\left|{a}_{i}\right|}^{2}}} $$

The TARC for a two-port antenna system is given as^28^

10 $${\Gamma }_{a}^{t}= \frac{\sqrt{\left({\left|{S}_{11}+{S}_{12}{e}^{j\theta }\right|}^{2}\right)+\left({\left|{S}_{21}+{S}_{22}{e}^{j\theta }\right|}^{2}\right)}}{\sqrt{2}}$$

**Figure 19:**
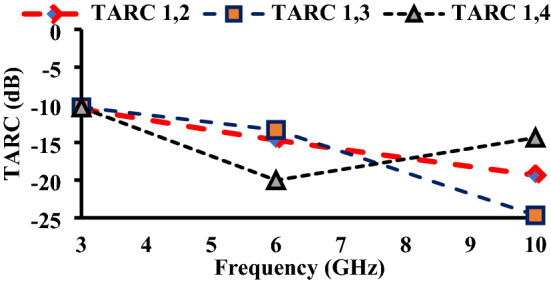
TARC of the proposed antenna.

The TARC value should be less than 0 dB. The TARC values of the proposed wristband antenna are less than − 10 dB as depicted in Fig. 19.

### Channel capacity loss (CCL)

The CCL gives information about the system’s channel capacity loss that occurred while the correlation effect was being performed^29^. It is calculated using the following matrix equation, which is derived from the reflection and transmission coefficients.

11 $$CCL= -{log}_{2}{\left|\psi \right|}^{R}$$

where $${\left|\psi \right|}^{R}= \left|\begin{array}{cc}{\rho }_{11}& {\rho }_{12}\\ {\rho }_{21}& {\rho }_{22}\end{array}\right|$$ and $${\rho }_{11 }= \left(1- {\left|{S}_{11}\right|}^{2}-{\left|{S}_{12}\right|}^{2}\right)$$, $${\rho }_{12}= -\left({S}_{11}*{S}_{12}+{S}_{21}*{S}_{22}\right)$$,$$\rho_{21} = - \left( {S_{22} *S_{21} + S_{12} *S_{21} } \right)$$, $$\rho_{22} = \left( {1 - \left| {S_{22} } \right|^{2} - \left| {S_{21} } \right|^{2} } \right)$$.

**Figure 20:**
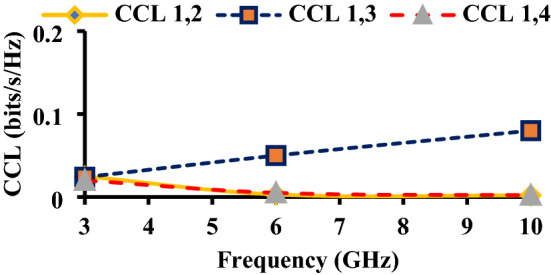
CCL of the proposed antenna.

The CCL should be less than 0.4 bits/s/Hz. The measured CCL values are less than 0.1 bits/s/Hz as shown in Fig. 20.

### SAR performance

**Figure 21:**
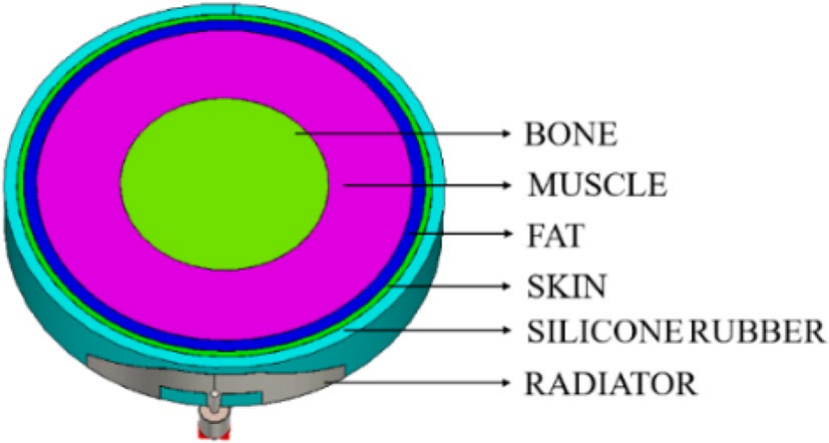
Cylindrical human body model for SAR calculation.

**Figure 22:**
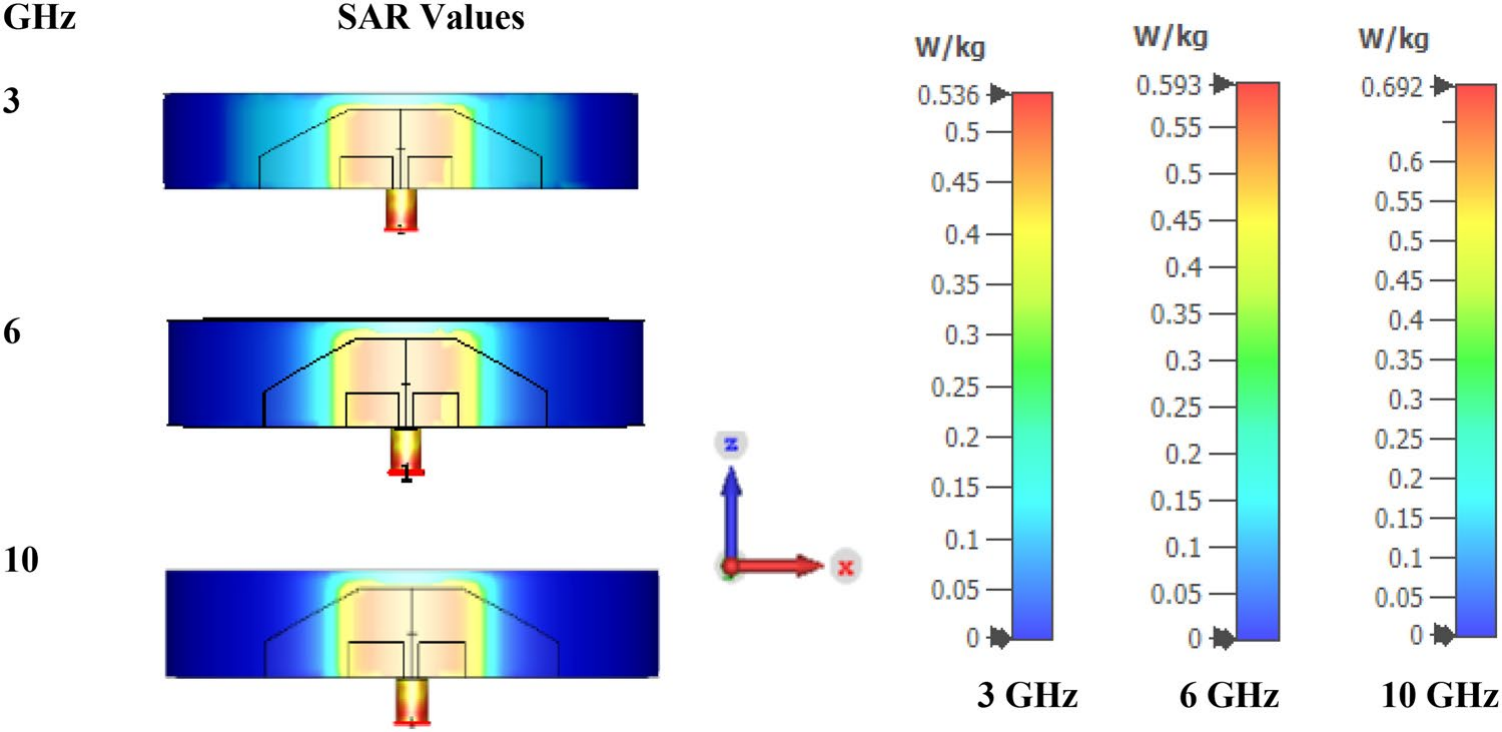
SAR performance of the antenna at different frequencies.

**Figure 23:**
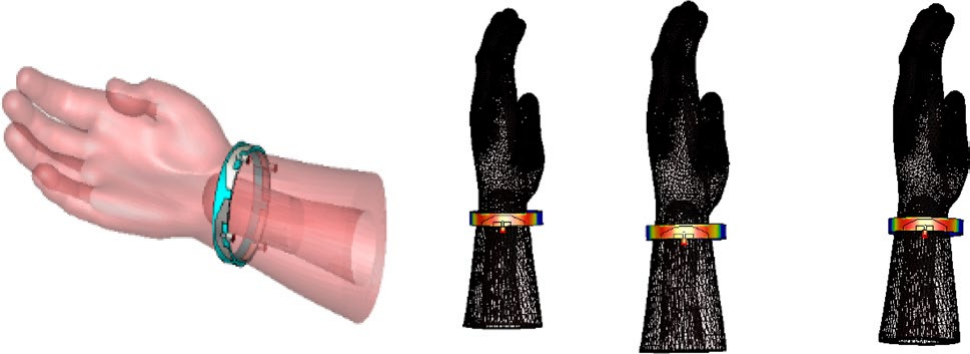
Imported human hand model and the proposed antenna.

SAR is the amount of radiation absorbed by human tissues when an antenna is kept in close proximity to the human body. The safer SAR limit set by the FCC and the International Commission on Non-Ionizing Radiation Protection (ICNIRP) is approximately 1.6 W/kg for 1 g of tissue and 2 W/kg for 10 g of tissue^30^. The skin, fat, muscle, and bone prototypes are designed for SAR calculations. The thicknesses of skin, fat, muscle, and bone are considered as 1 mm, 2 mm, 13 mm, and 16 mm, respectively. Table 1 shows the electrical properties of skin, fat, muscle, and bone at frequencies of 3 GHz, 6 GHz, and 10 GHz^31,32^. Since the substrate used in this work is a cylindrical wristband made of silicone rubber, a cylindrical human body model was chosen over a rectangular human body model. Figure 21 shows a schematic of the cylindrical human body model with skin, fat, muscle, and bone. The SAR values computed at the wrist at three different frequencies (3 GHz (starting), 6 GHz (middle), and 10 GHz (end)) are shown in Fig. 22. SAR values obtained are 0.536 W/kg, 0.593 W/kg, and 0.692 W/kg for 3 GHz, 6 GHz, and 10 GHz, respectively. It is found that all of the values are less than 1.6 W/kg. Also, the human wrist model is imported into CST and simulated by embedding the designed antenna into the hand model as shown in Fig. 23. The radiation performance of the hand model is also good in terms of SAR.

**Table 1:** Electrical properties of the human body tissues.

Tissues	Frequency (GHz)	Permittivity ($\varepsilon_{r}$)	Loss tangent ($\delta$)	Thermal conductivity (W/m/K)	Density (kg/${\text{m}}^{3}$)
Skin	3	37.358	0.2786	0.293	1100
	6	34.215	0.3584		
	10	30.705	0.4806		
Fat	3	5.2138	0.1501	0.201	910
	6	4.8608	0.1961		
	10	4.5572	0.2337		
Muscle	3	51.936	0.2480	0.53	1041
	6	47.069	0.3493		
	10	41.954	0.4647		
Bone	3	8.35	0.1434	0.2	1810
	6	7.73	0.173		
	10	7.6	0.196

**Figure 24:**
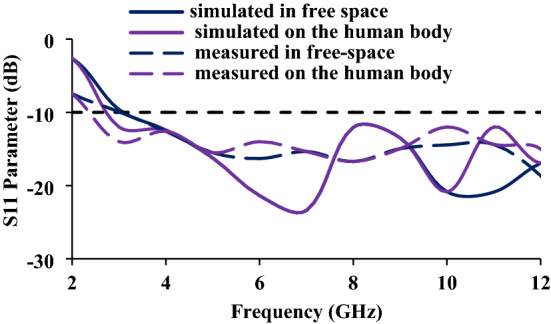
Reflection coefficients of the wristband antenna (in free space and on the human body).

The fabricated antenna prototype is measured, using the vector network analyzer, in both free space and on a human wrist, as shown in Fig. 9a, b, respectively. Figure 24 depicts the S-parameter curves for both free space and on-body conditions. It is clear from Fig. 9 that the S-parameters does not vary in free space or on human body. Therefore, the proposed antenna can be used for wearable applications.

Table 2 compares the proposed work to previously reported antennas in the literature. The comparison leads to the following conclusions:

- It is observed that the antennas reported in^8,10^, and^28,33,34^ are fabricated on rigid substrates, while^1–6^ and^35^ use flexible substrates like the proposed work.
- In this work, a wide impedance bandwidth of 9.35 GHz (2.75–12 GHz) is achieved, and four antenna elements are integrated over the flexible silicone rubber substrate, which is distinct from other flexible MIMO antennas reported.
- Isolation of more than 20 dB is observed across the entire operating band.
- The proposed antenna consists of a small size radiator of 0.09 $\lambda_{0}$ × 0.36 $\lambda_{0}$, where $\lambda_{0}$ corresponds to the lowest operating wavelength.
- A simple fabrication technique is used in comparison to^35^, which includes three processing steps for placing antennas on the substrate material.

**Table 2:** Comparison of proposed work with the reported antennas.

Refs	Frequency (GHz)	Substrate	Antenna size (*l* ${\text{mm}} \times$ *w* mm $\times$ *h* mm) (in $\lambda_{0}$)	Bandwidth (GHz)	No of antenna elements	Isolation (dB)
Ref.^1^	1.8, 2.45	Jean	124 $\times$ 90 $\times$ 1 (0.74 $\lambda_{0} \times$ 0.54 $\lambda_{0} \times$ 0.01 $\lambda_{0} )$	1.78–1.98 2.48–2.505	1	–
Ref.^2^	2.6	Taconic	250 × 200 × 1.6 (2.2 $\lambda_{0}$ × 1.7 $\lambda_{0}$ × 0.013 $\lambda_{0} )$	2.57–2.647	3	< 20
Ref.^3^	1.45	PDMS	85 $\times$ 60 $\times$ 0.8 (0.33 $\lambda_{0} \times$ 0.23 $\lambda_{0} \times$ 0.003 $\lambda_{0}$)	1.16–1.94	4	< 15
Ref.^4^	2.45, 2.5	NinjaFlex	54 × 65 × 1.2 (0.35 $\lambda_{0}$ × 0.4 $\lambda_{0}$ × 0.01 $\lambda_{0}$)	1.94–2.93	1	–
Ref.^5^	1–8	Kapton	48 × 34.9 × 0.13 (0.2 $\lambda_{0}$ × 0.12 $\lambda_{0}$ × 0.0004 $\lambda_{0}$)	1–8	1	–
Ref.^6^	0.9, 1.8	Jean	240 × 240 × 1 (0.76 $\lambda_{0}$ × 0.76 $\lambda_{0}$ × 0.003 $\lambda_{0} )$	0.95–0.98 1.8–1.85	1	–
Ref.^8^	5.8	FR-4	29 × 48 × 1.6 (0.52 $\lambda_{0}$ × 0.9 $\lambda_{0}$ × 0.03 $\lambda_{0} )$	5.27–6.27	3	> 15
Ref.^10^	2.93–20	FR-4	18 × 34 × 1.6 (0.18 $\lambda_{0}$ × 0.3 $\lambda_{0}$ × 0.02 $\lambda_{0} )$	2.93–20	2	22
Ref.^28^	5.8	FR-4	25 × 30 × 1.524 (0.4 $\lambda_{0}$ × 0.5 $\lambda_{0}$ × 0.03 $\lambda_{0} )$	5.4–6.1	2	13
Ref.^33^	5.4, 6	FR-4	30 × 30 × 1.6 (0.54 $\lambda_{0}$ × 0.54 $\lambda_{0}$ × 0.03 $\lambda_{0} )$	5.4–6	4	18
Ref.^34^	3.5	FR-4	40 × 100 × 0.8 (0.5 $\lambda_{0}$ × 1.15 $\lambda_{0 }$ × 0.01 $\lambda_{0} )$	3.47–3.57	8	< 15
Ref.^35^	1.92–2.17	Nelco NY9220	20 × 20 × 0.508 (18.7 $\lambda_{0}$ × 18.7 $\lambda_{0}$ × 0.47 $\lambda_{0}$)	27–29.5	2	< 15
Ref.^36^	2.4, 5.8	PLA	10 × 190 × 3 (0.075 $\lambda_{0}$ × 1.4 $\lambda_{0}$ × 0.02 $\lambda_{0} )$ Unit cell radiator 9 × 42.4 ${\text{ mm}}^{2}$ (0.067 $\lambda_{0}$ × 0.316 $\lambda_{0} )$	2.24–3.14, 4.61–7	3	> 15
This work	3.1–10.6	Silicone rubber	12 × 202 × 2 (1.1 $\lambda_{0}$ × 18.4 $\lambda_{0}$ × 0.18 $\lambda_{0} )$ Unit cell radiator 10 × 40 ${\text{mm}}^{2}$ (0.09 $\lambda_{0}$ × 0.36 $\lambda_{0}$)	2.75–12	4	> 25

## Conclusion

This paper presents the design and fabrication of a silicone rubber-based quad-port wristband wearable antenna. The diversity performance (pattern) is achieved by arranging four identical antenna elements in the horizontal plane with equal inter-element spacing. The simulated and measured results are found in the agreement. A low correlation value (of < 0.18) is achieved. Other diversity metrics, such as ADG, EDG, TARC, and CCL, are calculated and met the specifications. The wristband antenna offers a wide impedance bandwidth of 9.35 GHz, gain of 3.41 dB, and an efficiency of 89.3%. With a SAR value of 0.536 W/kg per 1 g of tissue, the proposed wristband antenna is suitable for on-body communication, and a promising candidate for tracking, monitoring, and security applications.

## Data Availability

The datasets generated during and/or analysed during the current study are available from the corresponding author on reasonable request.
